# Deletion of the phosphatase INPP5E in the murine retina impairs photoreceptor axoneme formation and prevents disc morphogenesis

**DOI:** 10.1016/j.jbc.2021.100529

**Published:** 2021-03-10

**Authors:** Ali S. Sharif, Cecilia D. Gerstner, Martha A. Cady, Vadim Y. Arshavsky, Christina Mitchell, Guoxin Ying, Jeanne M. Frederick, Wolfgang Baehr

**Affiliations:** 1 Department of Ophthalmology, University of Utah Health Science Center, Salt Lake City, Utah, USA; 2 Department of Ophthalmology, Duke University, Durham, North Carolina, USA; 3 Department of Biochemistry and Molecular Biology, Monash University, Clayton, Victoria, Australia; 4 Department of Neurobiology & Anatomy, University of Utah, Salt Lake City, Utah, USA; 5 Department of Biology, University of Utah, Salt Lake City, Utah, USA

**Keywords:** INPP5E, Joubert syndrome, rod and cone photoreceptors, axoneme, disc morphogenesis, connecting cilium, retina degeneration, CC, connecting cilia, CETN2, centrin-2, CO, Covance, Inc, COS, cone outer segment, ERG, electroretinography, IFT, intraflagellar transport, INPP5E, phosphatidylinositol polyphosphate 5-phosphatase, IS, inner segment, JBTS, Joubert syndrome, ONL, outer nuclear layer, OS, outer segment, PFA, paraformaldehyde, PIP_2_, PI (4, 5)P_2_, PI4P, phosphoinositol-4-phosphate, PT, Proteintech, RIS, rod inner segment, ROS, rod outer segments

## Abstract

INPP5E, also known as pharbin, is a ubiquitously expressed phosphatidylinositol polyphosphate 5-phosphatase that is typically located in the primary cilia and modulates the phosphoinositide composition of membranes. Mutations to or loss of INPP5E is associated with ciliary dysfunction. INPP5E missense mutations of the phosphatase catalytic domain cause Joubert syndrome in humans—a syndromic ciliopathy affecting multiple tissues including the brain, liver, kidney, and retina. In contrast to other primary cilia, photoreceptor INPP5E is prominently expressed in the inner segment and connecting cilium and absent in the outer segment, which is a modified primary cilium dedicated to phototransduction. To investigate how loss of INPP5e causes retina degeneration, we generated mice with a retina-specific KO (*Inpp5e*^F/F^;Six3Cre, abbreviated as ^ret^*Inpp5e*^−/−^). These mice exhibit a rapidly progressing rod–cone degeneration resembling Leber congenital amaurosis that is nearly completed by postnatal day 21 (P21) in the central retina. Mutant cone outer segments contain vesicles instead of discs as early as P8. Although P10 mutant outer segments contain structural and phototransduction proteins, axonemal structure and disc membranes fail to form. Connecting cilia of ^ret^*Inpp5e*^−/−^ rods display accumulation of intraflagellar transport particles A and B at their distal ends, suggesting disrupted intraflagellar transport. Although INPP5E ablation may not prevent delivery of outer segment–specific proteins by means of the photoreceptor secretory pathway, its absence prevents the assembly of axonemal and disc components. Herein, we suggest a model for INPP5E–Leber congenital amaurosis, proposing how deletion of INPP5E may interrupt axoneme extension and disc membrane elaboration.

INPP5E is a farnesylated phosphatidylinositol polyphosphate 5-phosphatase (1, 2) catalyzing the hydrolysis of the 5 phosphate from PI (4,5)P_2_ (PIP2), and PI (3,4,5)P_3_ (reviewed in (3, 4)). Phosphatidylinositol polyphosphates participate in cell division, integral membrane protein transport, vesicular trafficking, and cilia formation and ciliary function (4, 5, 6). INPP5E is associated with Joubert syndrome (JBTS) and mental retardation, truncal obesity, retinal dystrophy, and micropenis syndrome (7, 8) caused by missense mutations in its phosphatase domain. JBTS is a syndromic ciliopathy affecting the brain, eyes, kidneys, and liver (9, 10) and presenting with ataxia, hyperpnea, polydactyly, molar tooth sign in the brain and Retinitis pigmentosa or Leber congenital amaurosis.

Ten phosphatidylinositol 5-phosphatases (INPP5A, B, D (SHIP), E, F, G (synaptojanin 1), H (synaptojanin 2), J, K (SKIP), and INPPL1) are present in mammals, and of these, three (A, B, and E) are farnesylated (11). Germline mouse KOs of INPP5E and INPP5K in mice are embryonically lethal, suggesting nonredundant roles for some phosphatase isoforms in various cells or subcompartments (7, 9, 12). *Inpp5e*^−/−^ mice (deletion of exons 7 and 8) died soon after birth; E18.6 *Inpp5e*^−/−^ embryos showed developmental arrest at the optic vesicle stage before appearance of the optic cup. *Inpp5e*^−/−^ E18.5 embryos displayed multiple cysts, polydactyly, and skeletal abnormalities (7). *Inpp5e*^−/−^ embryos were anophthalmic, suggesting severe consequences of INPP5E deletion during early eye development (7). A second *Inpp5e*^−/−^ mouse model (deletion of exons 2–6) confirmed the JBTS phenotype and identified disordered sonic hedgehog–dependent patterning during embryonic development (12). Conditional deletion of INPP5E in kidneys resulted in severe polycystic kidney disease and hyperactivation of PI3K/Akt and mTORC1 signaling (13). Similarly, deletion of the Inpp5e gene in the zebrafish led to cystic kidneys (14).

Using transfection of IMCD3 cells with plasmids encoding FLAG–INPP5E or EGFP–INPP5E, INPP5E localized predominantly in the primary cilia (15, 16). As a C-terminally farnesylated protein, INPP5E is chaperoned to the cilium by the prenyl-binding protein PDEδ (16, 17, 18). Ciliary localization in hTERT-RPE1, 293T, and IMCD3 cells was confirmed using a polyclonal anti-INPP5E antibody (14, 19, 20, 21). *Inpp5e*^−/−^ mouse embryonic fibroblasts developed primary cilia, suggesting that INPP5E activity is not required for ciliogenesis, but mutant cilia were more sensitive to resorption during the cell cycle (5, 6, 9). In contrast to primary cilia, EGFP–INPP5E introduced to rod photoreceptors by neonatal electroporation distributed to inner segments (ISs) and partially colocalized with the Golgi apparatus (22). Immunolabeling of dissociated rods similarly showed prominent IS signal for INPP5E and an additional nonuniform signal of the outer segment (OS) (23).

In this study, we confirm that INPP5E localizes to the WT photoreceptor IS by multiple means. We also deleted INPP5E from the retina by mating *Inpp5e*^F/F^ mice (12) with Six3Cre transgenic mice and observed that ^*ret*^*Inpp5e*^−/−^ rod outer segments (ROSs) initiate degeneration by postnatal day 10 (P10). While P10 connecting cilia (CC) of P10 mutant rods are nearly normal in length, shortened ROS are devoid of recognizable discs. Intraflagellar transport (IFT)-A and IFT-B particles accumulate in the proximal ^*ret*^*Inpp5e*^−/−^ OS, suggesting defective IFT, a bidirectional transport system powered by molecular motors (24). Thus, deletion of INPP5E primarily impairs axoneme extension and disc morphogenesis of both rods and cones.

## Results

### Generation of retina-specific *Inpp5e* KO mice

**Figure 1:**
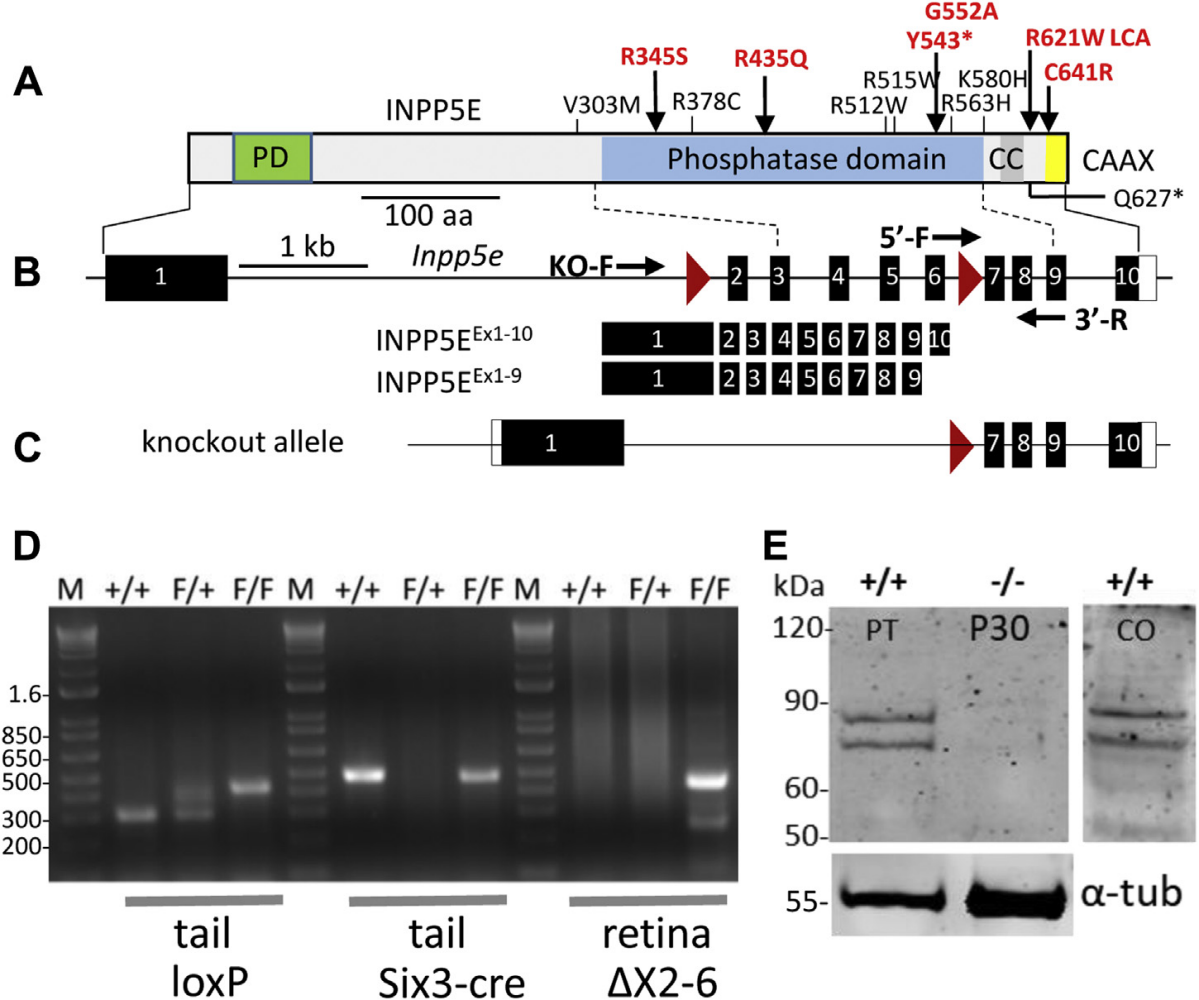
**Retina-specific KO of INPP5E.***A*, INPP5E major functional domains. Approximate positions of missense mutations associated with Joubert syndrome are indicated; mutations shown in *red* display a retina phenotype. *B*, the murine *Inpp5e* gene with 10 exons (*black bars*) and two LoxP sites (*red triangles*). Mouse *Inpp5e* gene expresses two splice variants, both isoforms contain the phosphatase domain. The shorter variant lacks the last exon carrying the CAAX motif. *C*, KO allele in which exons 2 to 6 are deleted. *D*, PCR genotyping protocol. The assay for WT and floxed alleles (*left*), for the absence or presence of Six3Cre (*middle*) and for deletion of exons 2 to 6 (*right*). Amplicons of lanes 4, 8, and 12 indicate a KO mouse. *E*, INPP5E immunoblot with commercially available antibody (Proteintech) (at P30) and custom antibody (Covance) (at P21), revealing the presence of two variants INPP5E^Ex1-10^ and INPP5E^Ex1-9^ in the mouse retina. Both variants were deleted in the KO. CAAX, motif for prenylation; CC, coiled-coil domain; INPP5E, phosphatidylinositol polyphosphate 5-phosphatase; P21, postnatal day 21; P30, postnatal day 30; PD, proline-rich domain.

INPP5E is a 72-kDa protein carrying a proline-rich domain in the N-terminal region, a large phosphatase active site encoded by exons 3 to 9, a coiled-coil domain, and a CAAX motif for C-terminal farnesylation (Fig. 1*A*). Mutations in human INPP5E associated with JBTS are located predominantly in the phosphatase domain. The mouse *Inpp5e* gene consists of 10 exons, producing two splice variants (Fig. 1*B*). The full-length variant is predicted to be farnesylated and membrane-anchored; the shorter variant lacking exon 10 and the CAAX box is predicted to be soluble.

To generate retina-specific KO (^ret^*Inpp5e*^−/−^) mice, *Inpp5e*^F/F^ mice were bred with Six3Cre mice (25). LoxP sites placed in introns 1 and 6 specify deletion of most of the phosphatase domain (Fig. 1*C*). LoxP, Six3Cre, and KO genotyping is shown (Fig. 1*D*). Immunoblotting with two independent antibodies against human INPP5E (Proteintech [PT]) and a mouse N-terminal fragment of INPP5E (custom-made at Covance, Inc. [CO]) confirmed the presence of two splice variants in WT retinal lysates and deletion of both isoforms in the conditional KO line (Fig. 1*E*).

### Electroretinography

**Figure 2:**
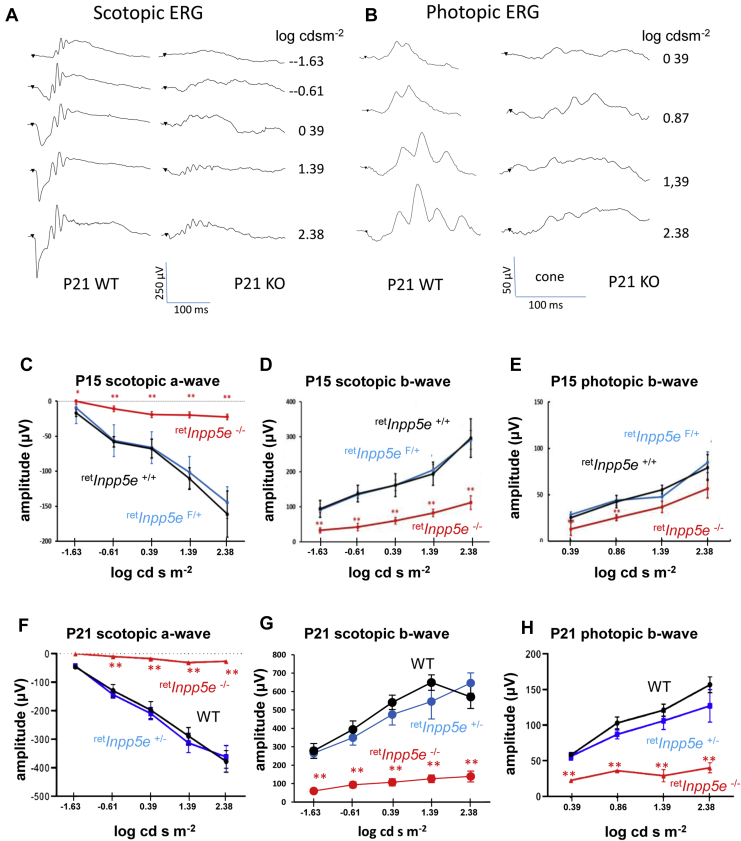
**Electroretinography of control and**^**ret**^***Inpp5e***^**−/−**^**mice.***A* and *B*, P21 scotopic (*A*) and photopic (*B*) ERG traces at various intensities. *C*–*H*, scotopic and photopic ERG measurements as a function of light intensity conducted at P15 (*C*–*E*) and P21 (*F*–*H*). A-wave and b-wave amplitudes were plotted as a function of flash intensity. Note the significant photopic b-wave activity of the ^ret^*Inpp5e*^−/−^ retina at P15 (*E*) and minor haploinsufficiency at P21 (*H*). P15 control, n = 7 and KO, n = 6. P21 control, n = 3 and KO, n = 4. Data were analyzed *via t* test: two samples assuming equal variance. Data are presented as the mean ±SEM. All a-wave scotopic amplitudes are less than ∗∗*p* < 0.01, and all b-wave amplitudes are less than ∗*p* < 0.05. Mutant mice were compared with their respective WT and heterozygous littermates. P15 control, n = 7 and KO, n = 6. P21, WT n = 4, heterozygous littermates, n = 3, and KO n = 4. There is no statistical significance at P15 photonic b-wave amplitude at 1.38 and 2.38 log cd s m^−2^. ERG, electroretinography; INPP5E, phosphatidylinositol polyphosphate 5-phosphatase; P15, postnatal day 15; P21, postnatal day 21.

We recorded whole-field scotopic (dark-adapted) and photopic (light-adapted) electroretinography (ERGs) at P15 and P21 at light intensities ranging from −1.63 to 2.38 log cd s/m^2^ (Fig. 2, *A* and *B*). Scotopic a-waves (Fig. 2, *C* and *F*) and scotopic b-waves (Fig. 2, *D* and *G*) of KO mice were barely recordable at P15 and P21. Scotopic a-waves of heterozygous littermates displayed normal a-wave amplitudes at P21 (Fig. 2*F*), indistinguishable from the WT, suggesting haplosufficiency. Surprisingly, the P15 ^ret^*Inpp5e*^−/−^ photopic ERG b-wave amplitudes were still recordable (Fig. 2*E*), presumably due in part to a slower degeneration of cones in the peripheral retina. At P21, ^ret^*Inpp5e*^−/−^ photopic ERG b-wave amplitudes were much reduced (Fig. 2*H*). The b-wave amplitudes of heterozygous littermates were somewhat reduced relative to the WT, but the reduction was statistically insignificant.

### ^ret^*Inpp5e*^−/−^ photoreceptors undergo rapid degeneration

**Figure 3:**
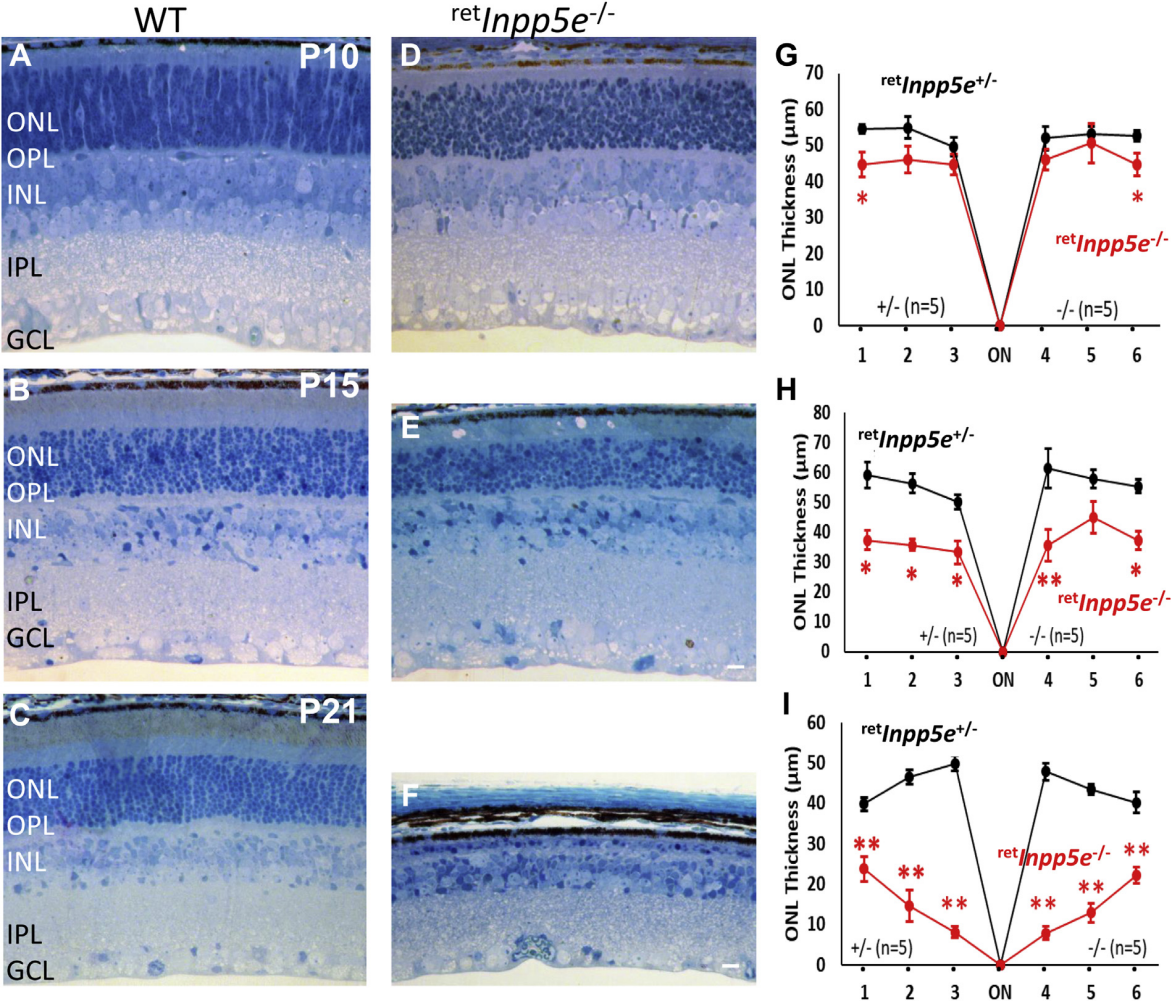
**Progressive photoreceptor degeneration in**^**ret**^***Inpp5e***^**−/−**^**mice.***A*–*F*, plastic sections obtained from the central retinas of WT (*A*–*C*) and ^ret^*Inpp5e*^−/−^ (*D*–*F*) mice at P10, P15, and P21. The ONL thickness in KO mice is nearly normal at P10 but is reduced to 7 to 8 rows of nuclei at P15. Only one nuclear row remains in the KO ONL at P21. The scale bar represents 10 μm. *G*–*I*, the ONL thickness of WT *versus* KO retinas, measured at 500 μm intervals from the optic nerve head (ON) at P10 (*G*), P15 (*H*), and P21 (*I*). Note that, at P21, the ^ret^*Inpp5e*^−/−^ ONL is preserved more at the periphery. Five control and KO animals were analyzed at each time point. Data are presented as the mean ±SEM. ∗*p*_0.05; ∗∗*p*_0.01. *n*, the number of animals analyzed; ONL, outer nuclear layer; P10, postnatal day 10; P15, postnatal day 15; P21, postnatal day 21.

Plastic sections of ^ret^*Inpp5e*^−/−^ and littermate WT control retinas collected at P10, P15, and P21 (Fig. 3, *A*–*F*) confirmed rapid photoreceptor degeneration, revealed by a progressively shrinking outer nuclear layer (ONL). At P10, ^ret^*Inpp5e*^−/−^ retinas (Fig. 3*D*) showed only a slightly reduced ONL thickness compared with the WT (Fig. 3*A*), but the distance between the retinal pigmented epithelium and ONL was substantially reduced, suggesting attenuation of the IS and/or OS volume. At P15, nuclear tiers were reduced by ∼30% (Fig. 3*E*), and at P21, only one row of nuclei, presumably cones, was present (Fig. 3*F*), consistent with rapid *rd1*-like degeneration. Evaluation of the ONL thickness across the entire retina (Fig. 3, G–I) showed 40% reduction in the central retina at P15 and nearly complete obliteration of the central ONL at P21. Persisting ONL thickness of the retinal periphery (Fig. 3*I*) is consistent with lower Cre expression in the peripheral retina under the Six3 promoter (25, 26). The ^ret^*Inpp5e*^−/−^ inner retina, including the inner nuclear and ganglion cell layers, was practically unaffected as judged by near-normal morphology of P10, P15, and P21 cryosections (Fig. 3, *D* and *E*).

### Photoreceptor INPP5E is an IS protein

**Figure 4:**
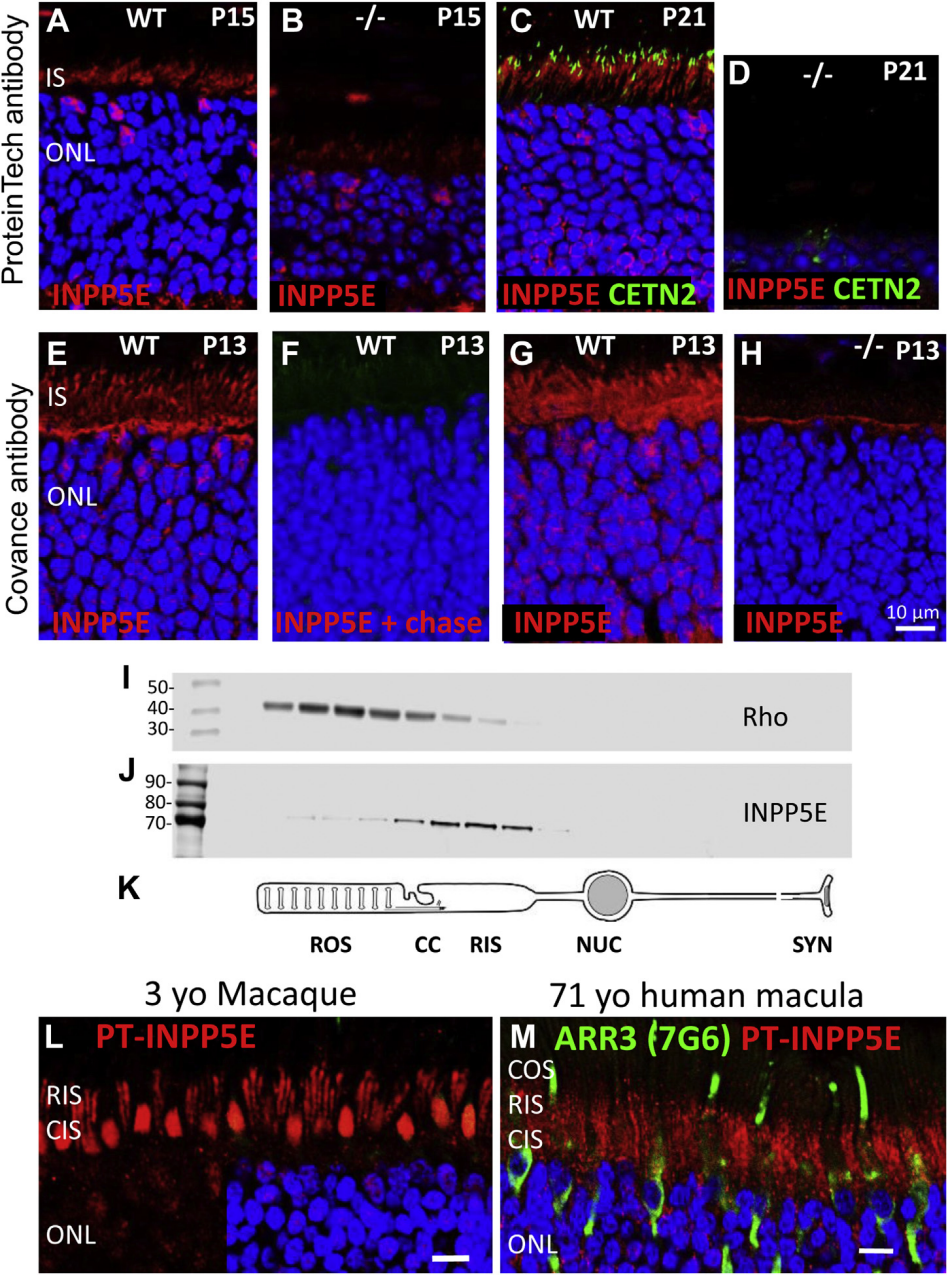
**INPP5E localization in rodent and primate photoreceptors.***A*–*D*, INPP5E immunostaining in WT (*A* and *C*) and ^ret^*Inpp5e*^−/−^ (*B* and *D*) retinal cryosections using the Proteintech antibody at P15 (*A* and *B*) and P21 (*C* and *D*). In ^ret^*Inpp5e*^−/−^ mice, INPP5E is weakly detectable at P15 and completely absent at P21. *E*–*H*, analysis of INPP5E custom-made Covance antibody specificity in P13 WT (*E*–*G*) and KO retina cryosections (*H*). *F*, the Covance antibody was preincubated with its cognate mouse N-terminal peptide, thereby preabsorbing reactivity and serving as a negative control. *I*–*K*, tangential sectioning, 5-μm-thick sections, of a rat retina. Proteins of individual sections were probed using anti-rhodopsin (4D2) (*I*) and anti-INPP5E (PT) (*J*) antibodies. *Rod cartoon* depicts approximate locations of individual sections (*K*). *L* and *M*, INPP5E immunolocalizations in 3-year-old (yo) macaque retina (*L*) and postmortem human macula from a 71-year-old (yo) donor (*M*). INPP5E (*red*) is detected robustly in cone (*bullet*-shaped profiles) and rod IS. In panel *M*, cones are identified by antibody recognizing primate cone arrestin (mAb 7G6, *green*) and nuclei are contrasted with DAPI (*blue*). INPP5E, phosphatidylinositol polyphosphate 5-phosphatase; P13, postnatal day 13; P15, postnatal day 15; P21, postnatal day 21.

INPP5E immunolocalization was performed using the well-characterized (PT) antibody raised against a His-tagged fusion protein corresponding to the N-terminal half of human INPP5E. WT cryosections analyzed at P15 (Fig. 4*A*) and P21 (Fig. 4*C*) revealed the presence of INPP5E in the IS with traces in the ONL, and absence in the OS. The INPP5E signal of the contemporaneously labeled KO IS was significantly reduced at P15 (Fig. 4*B*) and undetectable at P21 (Fig. 4*D*).

To verify specificity of the human antibody in mouse, we generated an independent antibody (CO) against the N-terminal peptide (MPSKSASLRHTEAC) of mouse INPP5E. As its sequence is distinct from the human homolog, the antibody is predicted to be mouse-specific. This antibody showed strong immunoreactivity in the entire IS, along the outer limiting membrane, with traces in the ONL (Fig. 4*E*); immunoreactivity was erased when the antibody was saturated with the antigenic peptide (Fig. 4*F*). Anti-INPP5E immunoreactivity was still detectable in KO sections at P15 with the PT antibody (Fig. 4*B*) and at P13 with the CO antibody (Fig. 4*H*), suggesting ineffective clearing of INPP5E or nonspecific binding. Differences in IS antigen localization using the PT antibody (fixed sections, Fig. 4*A*) and CO antibody (slightly fixed sections, Fig. 4*E*) are likely attributable to different fixation protocols (see Experimental procedures).

To further verify the IS localization of INPP5E, we performed the technique of combined serial tangential retina sectioning and immunoblotting (27). A Western blot of proteins from serial sections cut through a WT rat retina starting from the photoreceptor tips was probed with antibodies against rhodopsin (OS marker) and INPP5E (Fig. 4, *I* and *J*). This analysis revealed the presence of a single INPP5E species in sections corresponding to the IS (the rat *Inpp5e* gene expresses no splice variants) and only trace protein amounts in the sections representing the OS. A rod photoreceptor cartoon (Fig. 4*K*) indicates the approximate positions of the ROS, CC, rod inner segment (RIS), nucleus, and synapse.

The INPP5E localization was further confirmed in the retina of a macaque and macula of a 71-year-old human postmortem donor where INPP5E was detected in the IS of rods and cones (Fig. 4, *L* and *M*). In the perfusion-fixed macaque retina, INPP5E is prominent in the ellipsoid regions of the cone IS and RIS, respectively, with lesser amounts in perinuclear locales. Cone outer segment (COS) and ROS were mostly devoid of INPP5E. This pattern differs from INPP5E localization within the primary cilia where INPP5E is delivered with the aid of factors to achieve solubilization, PDEδ (PDE6D), and cargo displacement, ARL3–GTP (16).

### Localization of INPP5E to the CC

**Figure 5:**
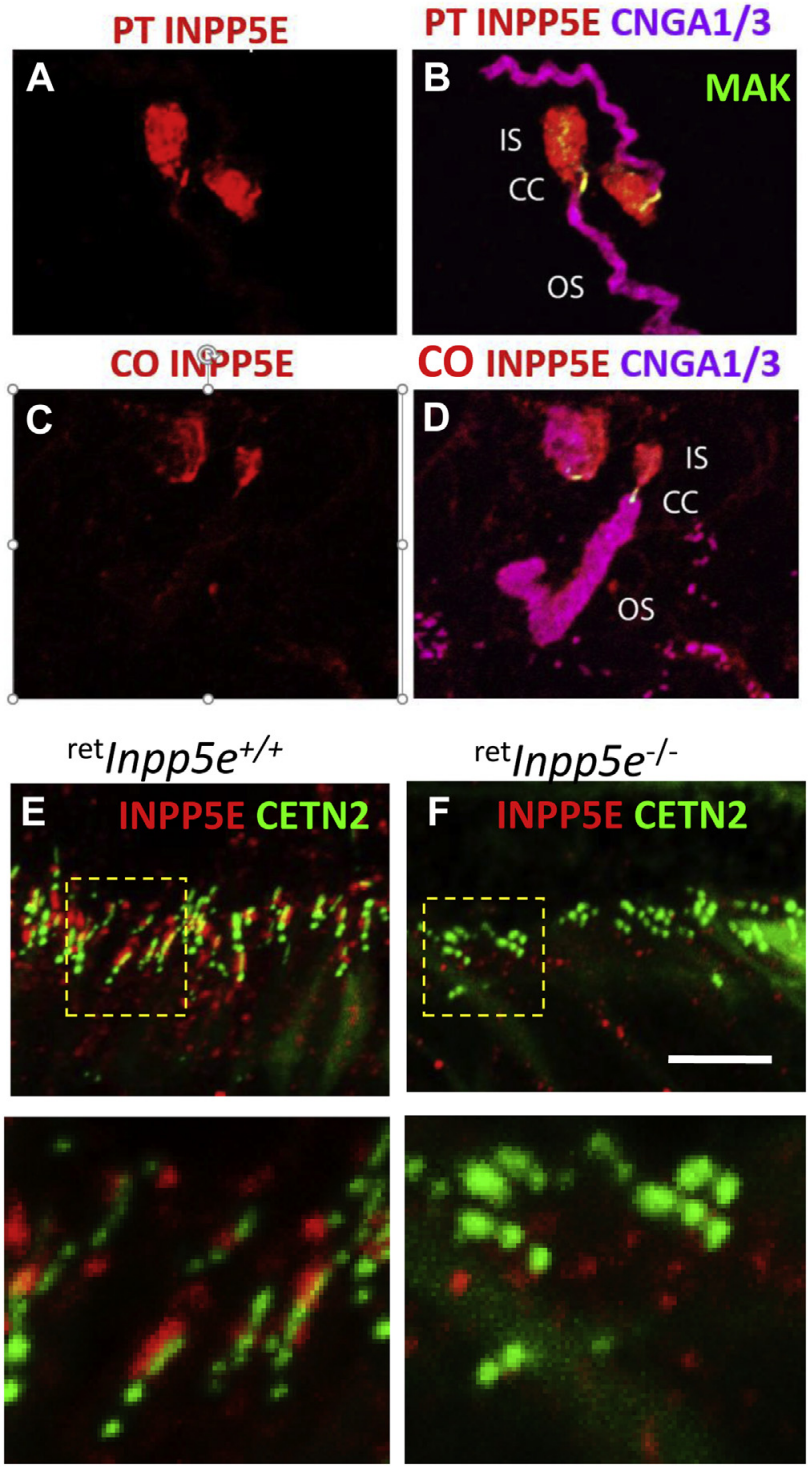
**INPP5E localizes at the CC.***A*–*D*, INPP5E distribution in isolated, lightly fixed mouse rods using the Proteintech (I and J) and Covance (K and L) antibodies (*red*). OS are labeled by an antibody against CNGA1/3 (*magenta*) and CC by an antibody against MAK (male germ cell–associated kinase, *green*). *E* and *F*, *Egfp-Cetn2*^+^; ^ret^*Inpp5e*^+/−^ (*E*) and *Egfp-Cetn2*^+^; ^ret^*Inpp5e*^−/−^cryosections (*F*) probed with anti-INPP5E (PT) (*red*). Underneath, enlargements of panels *E* and *F* as indicated. Note localization of INPP5E at the control CC and its absence in the *Inpp5e*^−/−^ CC. EGFP–CETN2 is represented by CETN2 (*green*). CC, connecting cilia; CETN2, centrin-2; INPP5E, phosphatidylinositol polyphosphate 5-phosphatase.

Dissociated mouse rods (23) were probed with both PT and CO antibodies (Fig. 5, *A*–*D*). The OSs were identified by anti-CNGA1/3 (cyclic nucleotide-gated channel subunits) located in the OS cell membrane and the CC by anti-MAK (male germ cell–associated kinase) antibodies localizing to the CC (28). These results clearly showed the absence of INPP5E in the OS and its prominent presence in both the IS and CC. Another CC marker is centrin-2 (CETN2), a Ca^2+^-binding protein associated with the CC and basal body. To demonstrate localization of INPP5E at the CC *in vivo*, we perfused P10 Egfp-Cetn2^+^^ret^*Inpp5e*^+/−^ and ^ret^*Inpp5e*^−/−^ mice with 4% paraformaldehyde (PFA), postfixed overnight, generated 100-μm retina sections, performed antigen retrieval with 1% SDS, and analyzed the sections treated with anti-INPP5E antibody by confocal microscopy. The results show the presence of INPP5E specifically at the ^ret^*Inpp5e*^+/−^CC (Fig. 5*E*) and the absence of INPP5E in ^ret^*Inpp5e*^−/−^ sections (Fig. 5*F*).

### Absence of PDEδ does not affect INPP5E localization

**Figure 6:**
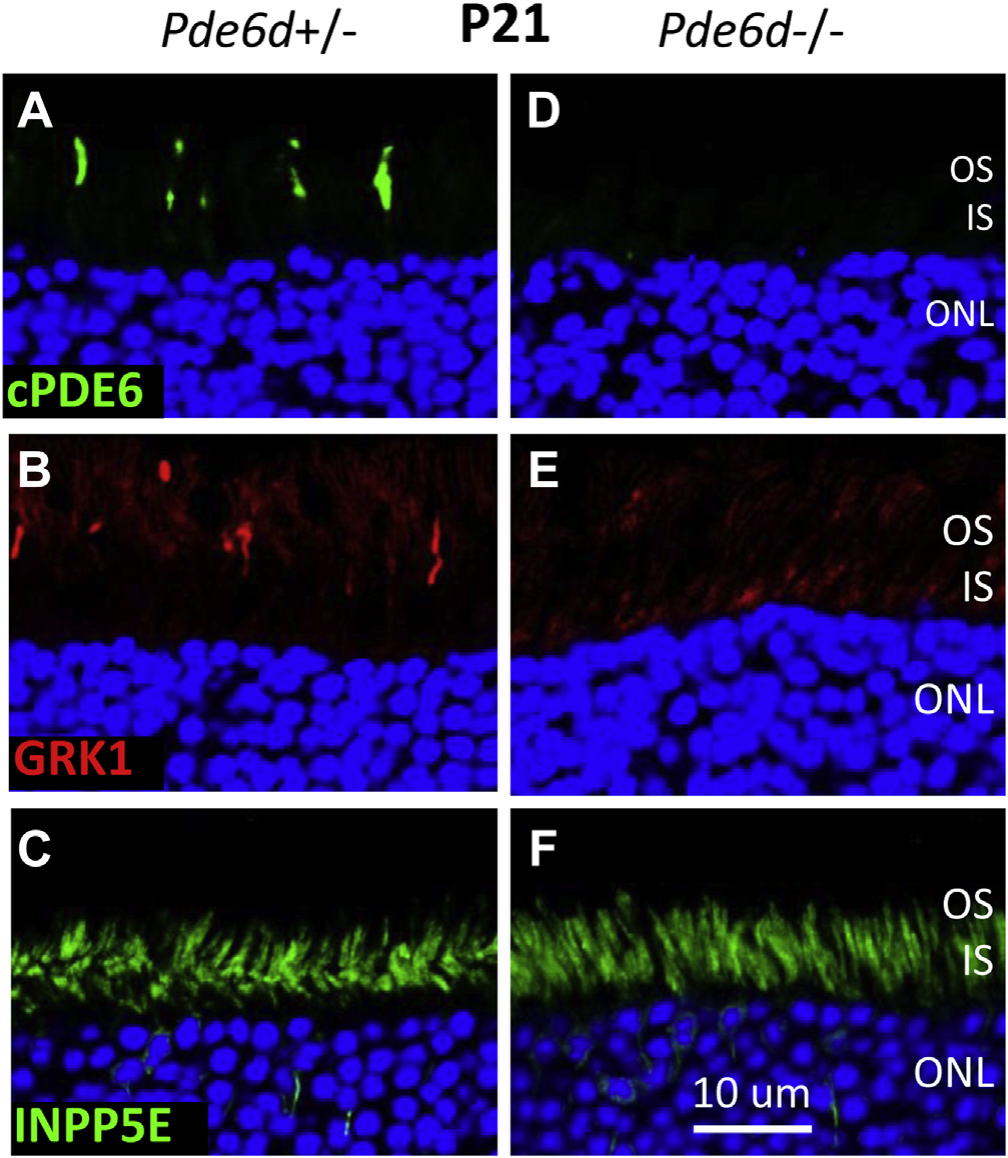
**INPP5E localization in photoreceptors is independent of PDEδ.***A*–*F*, *Pde6d*^+/−^ (*A*–*C*) and *Pde6d*^−/−^ cryosections (*D*–*F*) were immunostained with anti-cPDE6 (*A* and *D*; *green*), anti-GRK1 (*B* and *E*; *red*), and anti-INPP5E (PT) (*C* and *F*; *green*) antibodies. Prenylated cone PDE6 and GRK1 require PDEδ for OS localization. INPP5E inner segment localization is PDEδ-independent. The scale bar represents 10 μm. INPP5E, phosphatidylinositol polyphosphate 5-phosphatase; OS, outer segment; PT, Proteintech;

The IS location of INPP5E was unchanged in cryosections obtained from *Arl3*^−/−^ mice (22), suggesting that it is not delivered in a complex with the prenyl-binding protein PDEδ. We verified that cellular distribution of farnesylated INPP5E is not dependent on PDEδ by analyzing its localization patterns in *Pde6d*^−/−^ photoreceptors (Fig. 6). The OS of the PDE6D^+/-^ cones contain geranylgeranylated PDE6α’ (cone PDE6 α-subunit) (Fig. 6*A*) and farnesylated GRK1 (G-protein receptor kinase) (Fig. 6*B*) whose transport to COS is completely impaired in the absence of PDEδ. (Fig. 6, *D* and *E*). In contrast, the localization of farnesylated INPP5E was insensitive to the absence of PDEδ (Fig. 6, *C* and *F*).

### Rhodopsin and PDE6 in ^ret^*Inpp5e*^−/−^ retina

**Figure 7:**
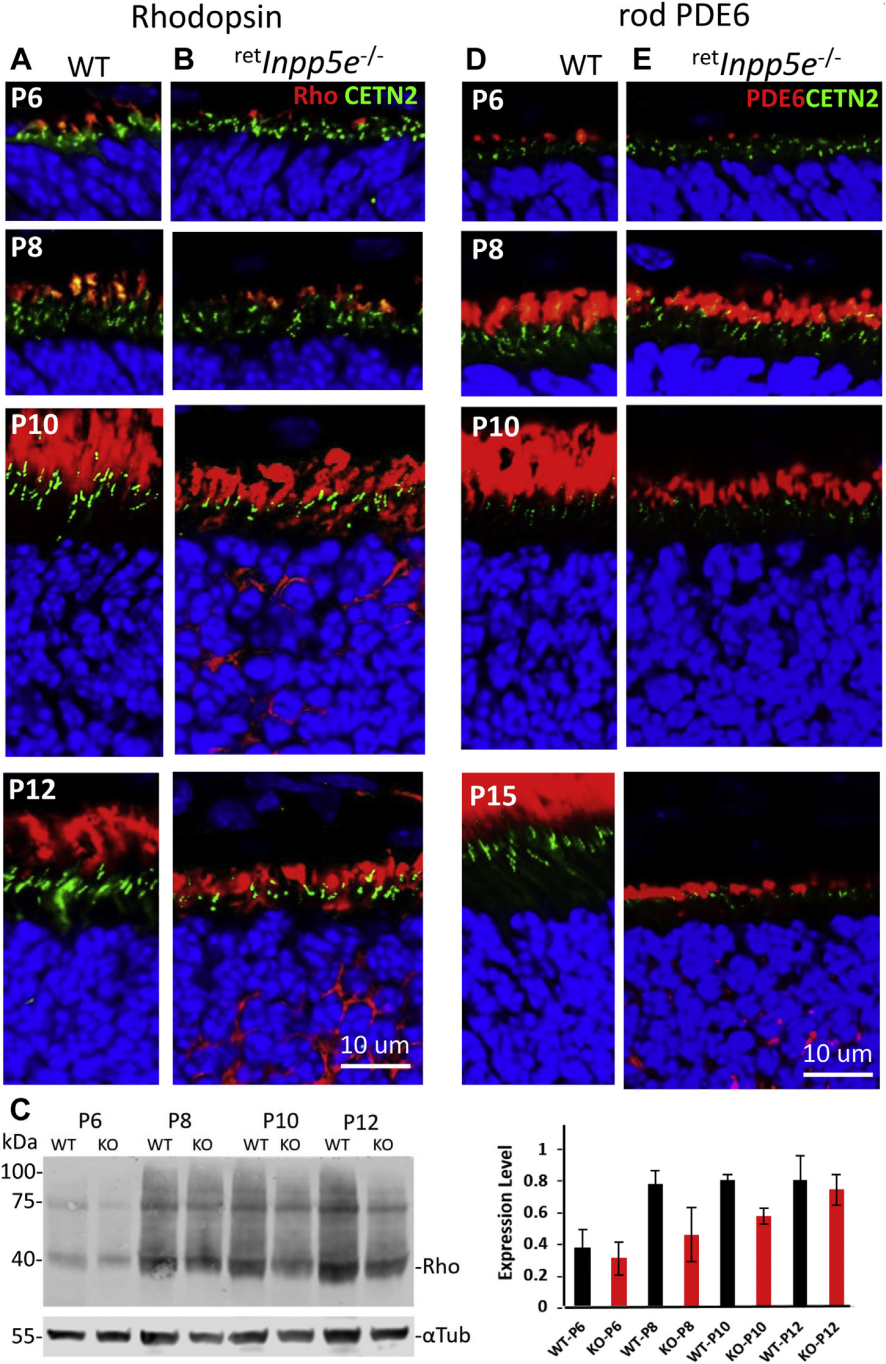
**Immunolocalization of rhodopsin and rod PDE6 at P6 – P15.***A* and *B*, cryosections from WT (*A*) and ^ret^*Inpp5e*^−/−^ (*B*) central retinas were probed with an anti-rhodopsin antibody (*red*) at P6, P8, P10, and P12. Transgenic EGFP–CETN2 (*green*) identifies centrioles and CC, and DAPI (*blue*) stains nuclei. Starting at P10, photoreceptor OS does not expand and begin to degenerate. The scale bar represents 10 μm. *C*, western blot of WT and KO retina lysates at P6-P12. Densities of rhodopsin bands (*left*) were normalized against the loading control α-tubulin and plotted on the graph on the *right*. *D* and *E*, cryosections from WT (*D*) and ^ret^*Inpp5e*^−/−^ (*E*) central retinas were probed with an anti-PDE6 antibody (MOE) at P6, P8, P10, and P15. Centrioles and CCs are identified by transgenically expressed EGFP–CETN2. Nuclei are labeled with DAPI.(4′,6-diamidino-2-phenylindole). CC, connecting cilia; CETN2, centrin-2; OS, outer segment; P6, postnatal day 6; P8, postnatal day 8; P10, postnatal day 10; P15, postnatal day 15.

In the WT and mutant retina at P6, the OS begin to expand and rhodopsin is present in nascent WT OS and smaller mutant OS (Fig. 7, *A* and *B*; P8 panels). At P10, mutant OSs are shorter than the corresponding WT OSs (Fig. 7, *A* and *B*; P10 panel) and rhodopsin begins to mislocalize and its CC (labeled with EGFP–CETN2) are located closer to the ONL, suggesting a shrinking, degenerating IS. At P12, the WT OSs are maturing, but the mutant OSs are stunted and rhodopsin accumulates throughout the ONL (Fig. 7, *A* and *B*, P12 panels). As judged by Western blotting (Fig. 7*C*, left panel), the level of rhodopsin in mutant rods is slightly reduced at P6 and about half at P10 and P12 (Fig. 7*C*, right panel).

Prenylated peripheral OS proteins (PDE6, GRK1) are thought to traffic the OS by diffusion, using PDEδ as a solubilization factor (29, 30) and ARL3–GTP as a cargo dispensation factor (22, 31, 32). In WT and ^ret^*Inpp5e*^−/−^ at P6, trace amounts of PDE6 are present in the OS (Fig. 7, *D* and *E*). At P8 (just after onset of degeneration), the level of OS PDE6 in ^ret^*Inpp5e*^−/−^ rods is comparable with the WT rods, but it is significantly reduced at P10 (Fig. 7*E*), consistent with the reduction of their OS size. These results suggest that endoplasmic reticulum docking, for post-translational modifications of PDE6, is not affected by INPP5E ablation.

### Mutant cones form spherical OSs

**Figure 8:**
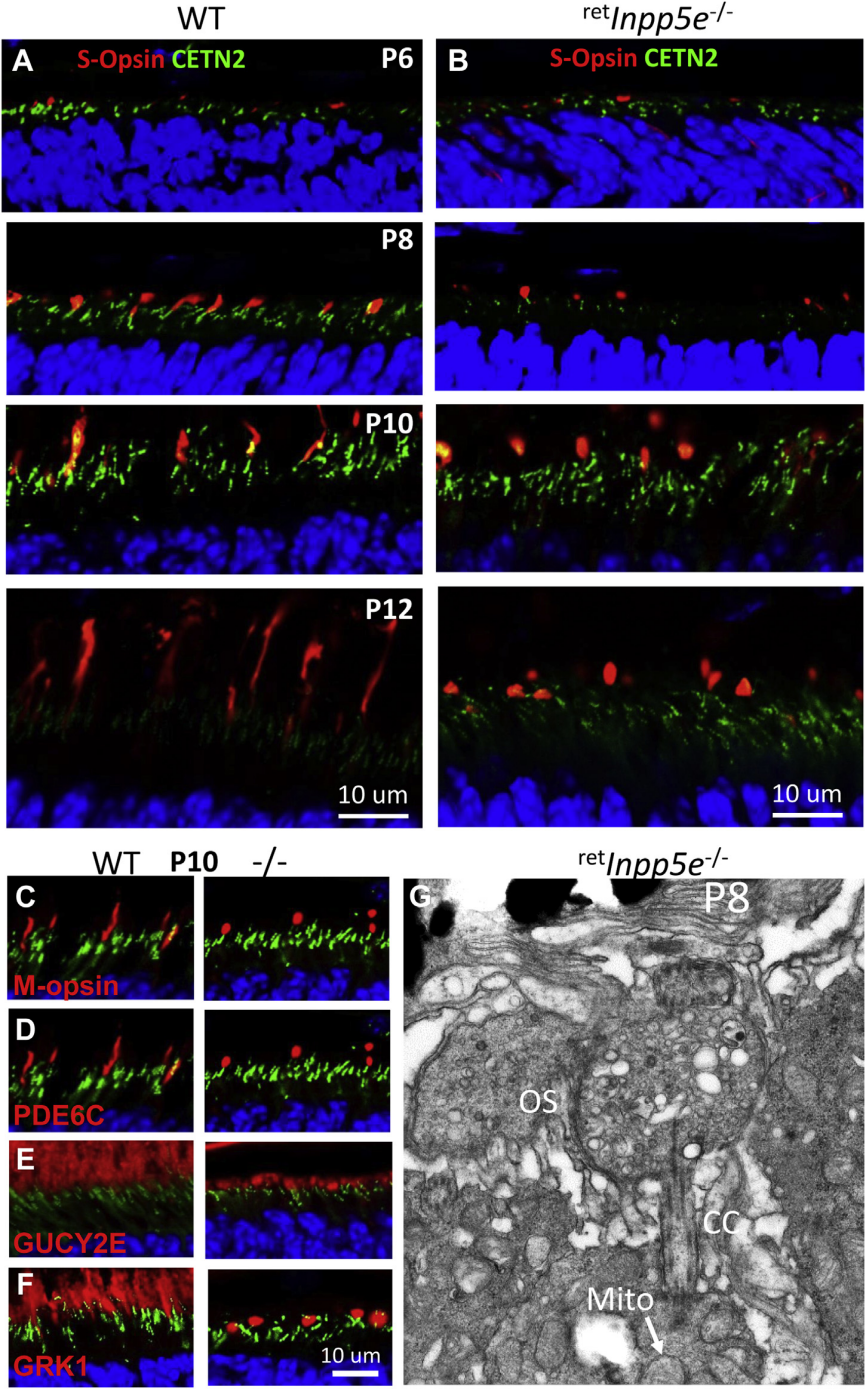
**Mutant cones form spherical OS.***A* and *B*, S-opsin localization in WT (*A*) and ^ret^*Inpp5e*^−/−^ (*B*) retinal cryosections at P6-P12. Cone OSs are labeled with an anti-S-opsin antibody. CCs are labeled by transgenically expressed EGFP–CETN2. In KO mice, S-opsin is located in the spherical cone OS at P8-P12. *C*–*F*, immunostaining of M-opsin (*C*), cone PDE6α’ (*D*), GC1 (*E*), and GRK1 (*F*) in WT (*left panels*) and mutant (*right panels*) retinas at P10. *G*, ultrastructure of ^ret^*Inpp5e*^−/−^ cone OS at P8. The OS appears as a spherical bag filled with vesicles and lacking OS axoneme extension and disc membranes. The identity of this cell as a cone derives from IS mitochondria (*white arrow*). See Ref. (53) for a representative image of a WT cone. CC, connecting cilia; CETN2, centrin-2; IS, inner segment; OS, outer segment; P6, postnatal day 6; P8, postnatal day 8; P10, postnatal day 10; P12, postnatal day 12.

The COSs begin to form at P4 (33) and, in the WT retina, can be detected by S-opsin immunofluorescence at P6 (Fig. 8*A*). At P8 to P10, WT COSs extend (Fig. 8*A*) while KO COSs form spherical structures (Fig. 8*B*). At P12, WT COSs are nearly mature while KO COSs remain spherical and increase in size. Similar spherical structures were seen when retinal sections were probed with antibodies against ML-opsin, cone PDE6, GRK1, and GUCY2E (mouse retina guanylate cyclase or GC1) (Fig. 8, *C*–*F*). Electron microscopy revealed that the mutant COS does not form discs, is filled with vesicles of various sizes, and contains rudimentary fragments of axonemal extension. Yet, the CC appears to be normal in length (Fig. 8*G*). Mitochondrion (white arrow) at the distal IS confirms the cell is a cone.

### Protein localization in the mutant OS

**Figure 9:**
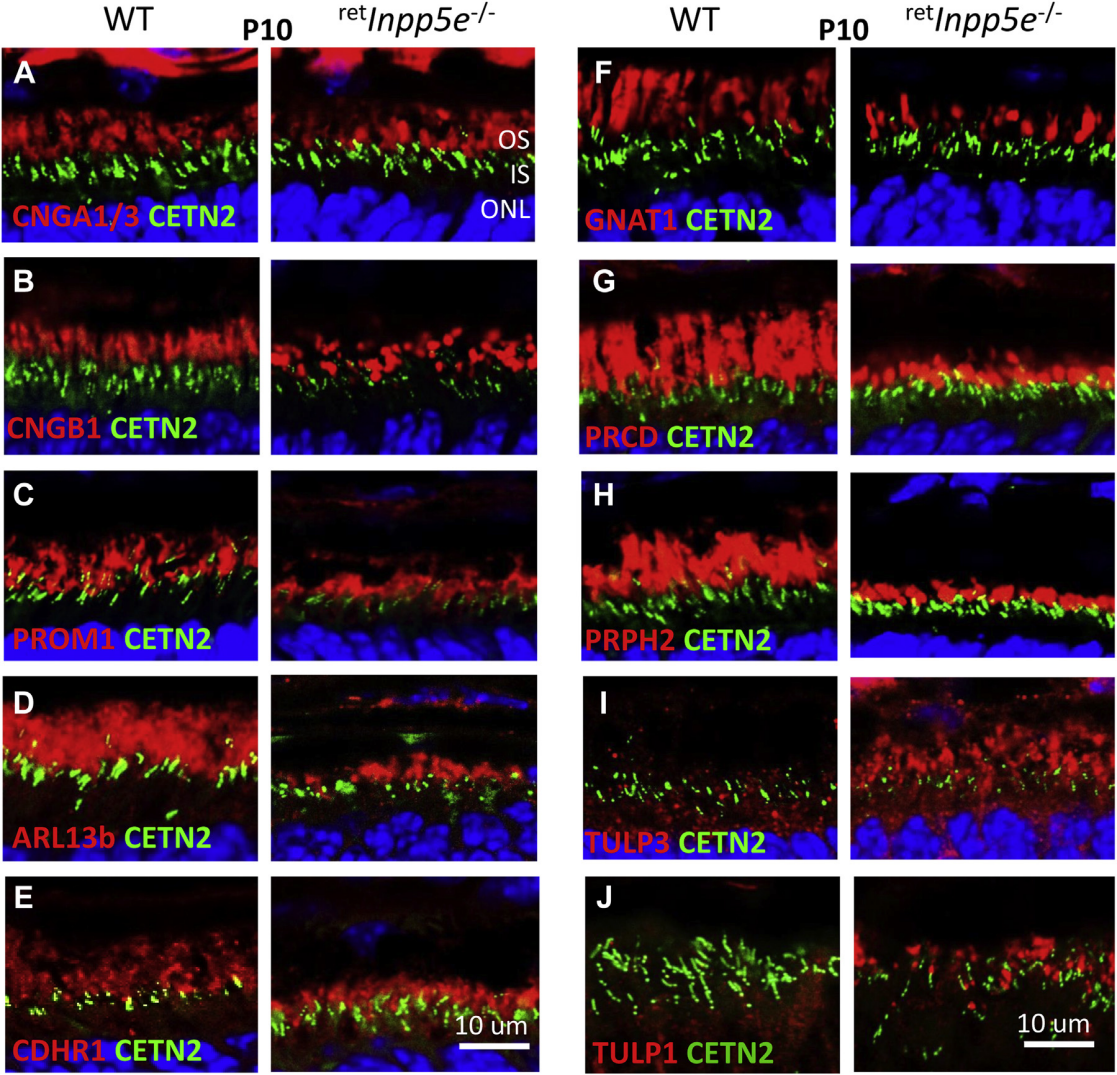
**Survey of OS proteins’ localization in WT and**^**ret**^***Inpp5e***^**−/−**^**retinas at P10.***A*–*I*, cryosections from WT (*left*) and KO (*right*) retinas were probed with antibodies against CNGA1/3 (*A*), CNGB1 (*B*), PROM1 (*C*), ARL13B (*D*), CDHR1 (*E*), GNAT1 (*F*), PRCD (*G*), PRPH2 (*rds*) (*H*), TULP3 (*I*) and TULP1 (*J*). The scale bar represents 10 μm. OS, outer segment; P10, postnatal day 10.

Immunolocalization of OS resident proteins was performed at P10 using a battery of well-characterized antibodies (22, 26) (Fig. 9). All investigated phototransduction proteins (GNAT1 (transducin α-subunit), PDE6 subunits, CNGA1/3 subunits) and structural proteins PRPH2 (peripherin-2), PROM1 (prominin 1), CDHR1 (Cadherin Related Family Member one alias protocadherin 21) were present in mutant ROS (Fig. 9). ARL13b (Arf-like protein 13b, INPP5E interactant) and PRCD (progressive rod-cone degeneration proteins), involved in packaging membranes during disc morphogenesis (34), were located normally as well. Exceptions were the tubby-like proteins TULP3 and TULP1 that were localized predominantly in the IS of WT rods (Fig. 9, I and *J*, left panels) but were additionally present in at much greater amount in the mutant OS (Fig. 9, *I* and *J*, right panels).

### Ultrastructure of the mutant ROS

**Figure 10:**
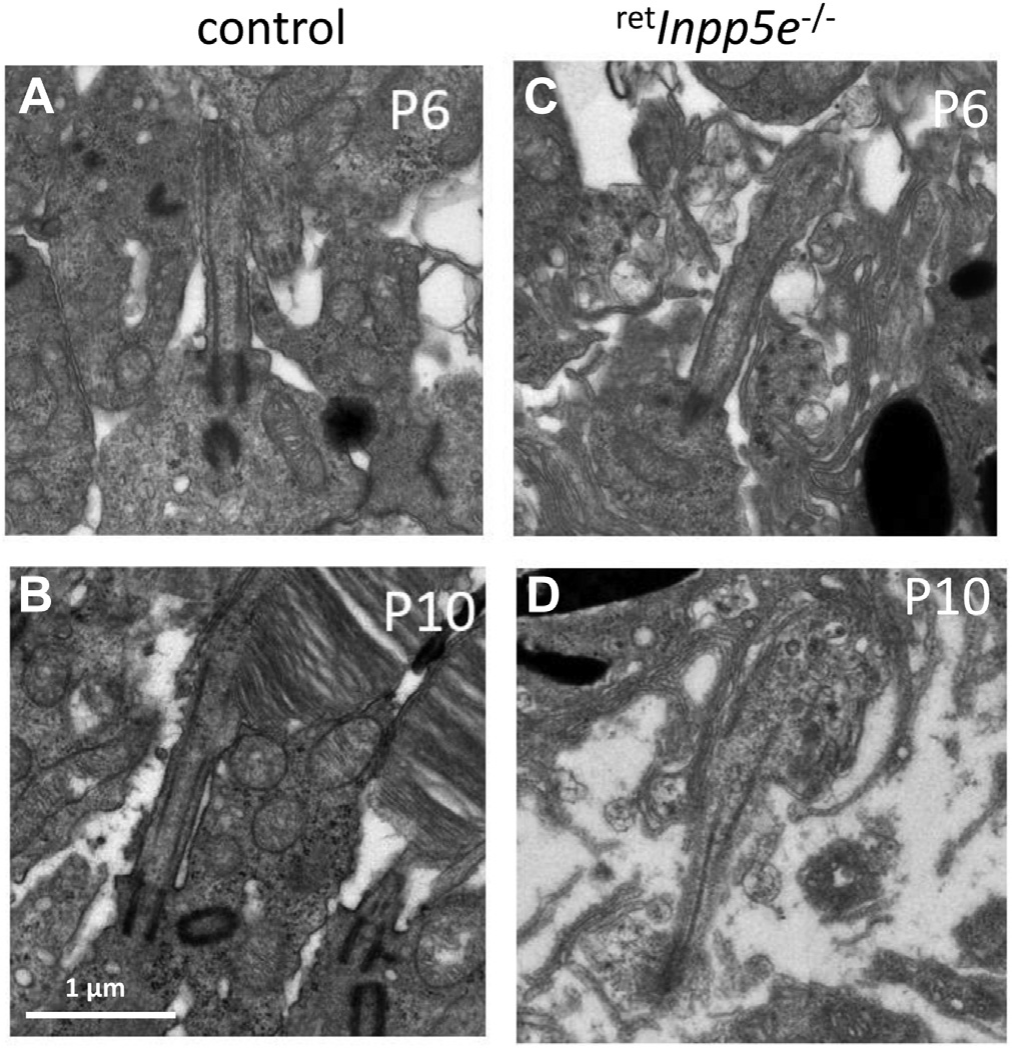
**Comparative ciliary and OS ultrastructure of WT and**^**ret**^***Inpp5e***^**−/−**^**rods.***A* and *C*, representative images of cilia emanating from WT (*A*) and ^ret^*Inpp5e*^−/−^ (*C*) rods at P6. *B* and *D*, representative images of WT (*B*) and ^ret^*Inpp5e*^−/−^ (*D*) rods at P10 illustrating abnormal transition zone structure and rudimentary OS without discs in the KO rod. OS, outer segment; P10, postnatal day 10.

We next explored the ultrastructure of WT and mutant rod photoreceptors using transmission electron microscopy. At P6, rods produce the primary cilium emanating from the basal body (Fig. 10*A*). The process of ciliogenesis is unaffected by the INPP5E KO (Fig. 10*C*). At P10, WT rods start forming the OS containing the regular disc structure (Fig. 10*B*). At this age, the CC structure of ^*ret*^*Inpp5e*^−/−^ rods remained normal and extended some axoneme fragments into the proximal OS, but a normal-size axoneme was not generated. Importantly, the membrane structure emanating from the CC was highly disorganized and failed to form an ordered stack of discs (Fig. 10*D*).

**Figure 11:**
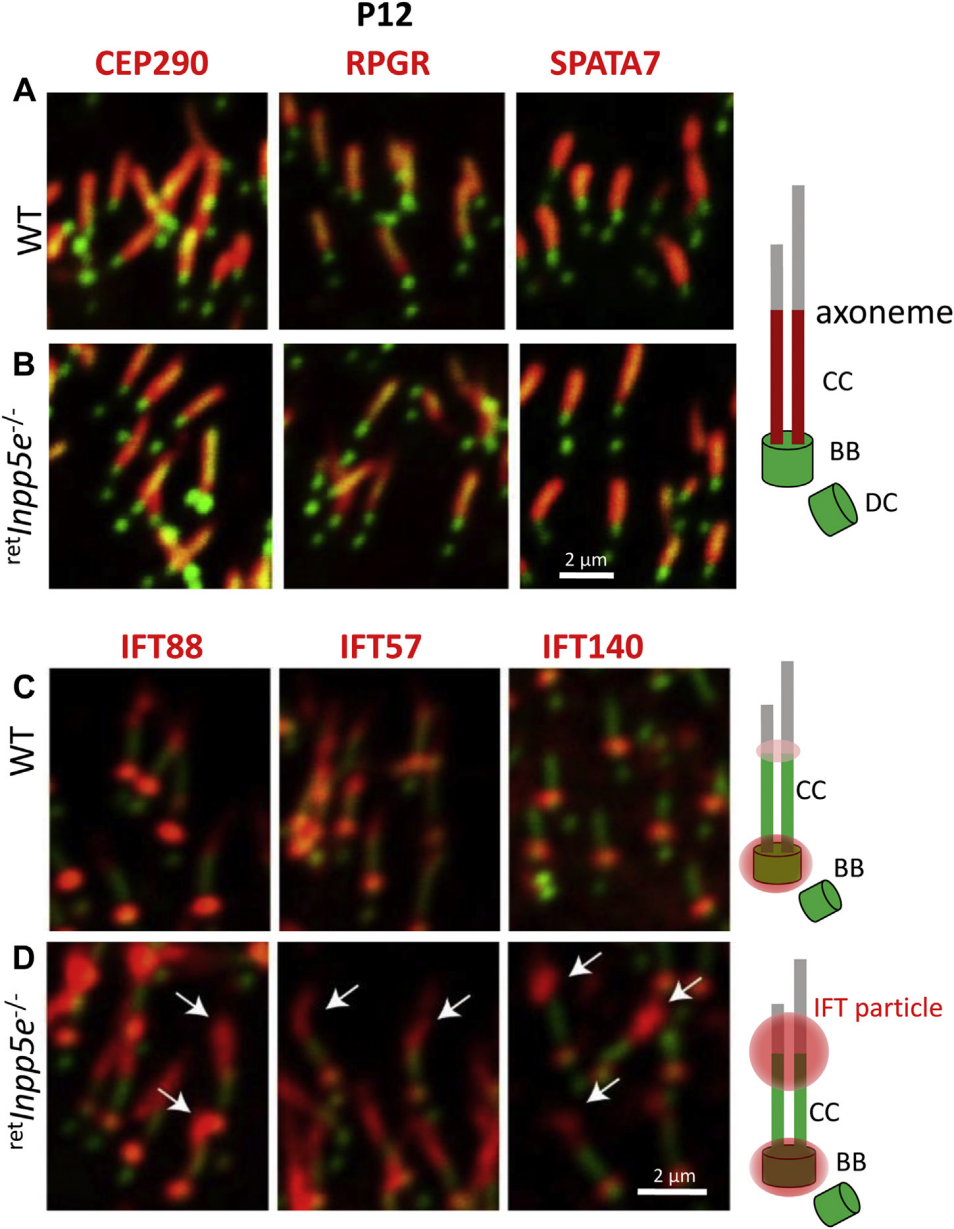
**Mislocalization of intraflagellar transport proteins and normal distribution of ciliary transition zone proteins in**^**ret**^***Inpp5e***^**−/−**^**rods.***A* and *B*, cryosections from WT (*A*) and ^ret^*Inpp5e*^−/−^ (*B*) retinas collected at P12 were probed with antibodies against CEP290, RPGR, and SPATA7 representing CC-specific proteins. Normal localization of these proteins suggests normal ultrastructure of the KO CC. The scale bar represents 2 μm. *C* and *D*, cryosections from WT (*C*) and ^ret^*Inpp5e*^−/−^ (*D*) retinas collected at P12 were probed with antibodies against IFT88, IFT57, and IFT140. In WT photoreceptors, IFT particles are concentrated next to the basal body. Low levels of IFT88 and IFT57 are also observed in the proximal OS. In ^ret^*Inpp5*e^−/−^ photoreceptors, a large fraction of IFT particles accumulates in the proximal OS (*arrows*). These changes are depicted in *cartoons* on the *right*. CC, connecting cilia; IFT, intraflagellar transport; OS, outer segment; P10, postnatal day 10; P12, postnatal day 12.

Immunolocalization conducted with retinal sections from WT and mutant mice (Fig. 11, *A* and *B*) demonstrated correct positions of CEP290 (centrosomal protein 290), RPGR (Retinitis Pigmentosa GTPase regulator), and SPATA7 (spermatogenesis-associated protein 7), suggesting that mutant CC is intact. Overall, the *Inpp5e*^−/−^ CCs appears to be indistinguishable from WT CC.

### Impairment of axoneme extension

IFT is a bidirectional protein transport pathway for proteins biosynthesized in the IS. IFT depends on molecular motors and IFT particles. We investigated the levels and positions of the IFT particles IFT88, IFT57, and IFT140 by immunostaining (Fig. 11*C*). IFT88 and IFT57 are subunits of the IFT-B particle, whereas IFT140 is a subunit of the IFT-A particle complex. Normally, IFT particles are strongly associated with the basal body and proximal OS and are essential for supporting anterograde and retrograde transport of the ciliary material (35, 36, 37). In P12 WT photoreceptors, IFT88 and IFT57 were strongly stained next to the basal body and weakly to the proximal OS, whereas IFT140 was seen only at the basal body (Fig. 11*C*). In KO photoreceptors, IFT88, IFT57, and IFT140 massively accumulated at the CC/OS junction and in the proximal mutant OS (Fig. 11*D*, white arrows), suggesting impaired retrograde IFT.

**Figure 12:**
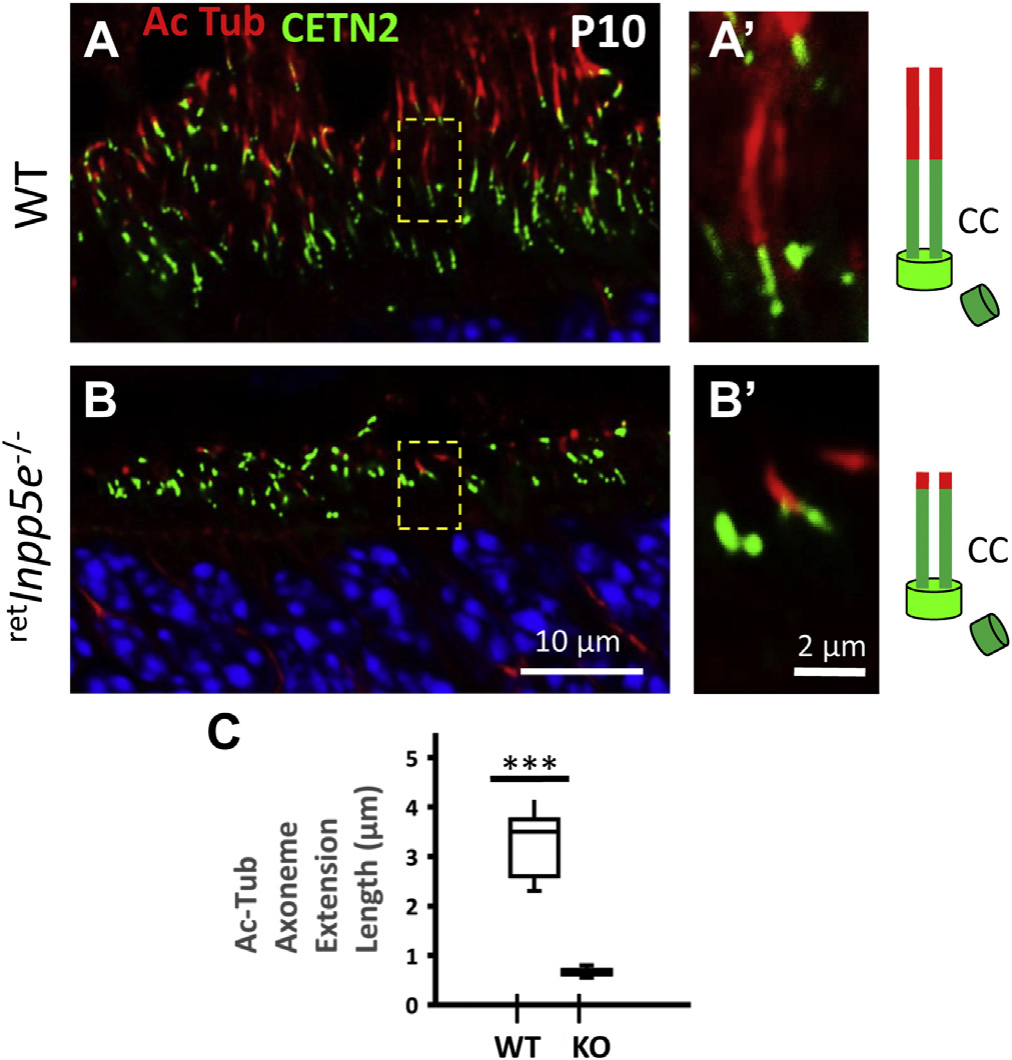
**Stunted axoneme in developing**^**ret**^***Inpp5e***^**−/−**^**photoreceptors.***A* and *B*, ^ret^*Inpp5e*^+/−^ (*A*) and ^ret^*Inpp5e*^−/−^ (*B*) retinal cryosections were stained with an antibody against acetylated α-tubulin (Ac-Tub). Panels *A’* and *B’* are enlargements. *Cartoons* illustrating basal bodies and CC (*green*) and axonemes (*red*) are shown, *right*. WT Ac-Tub positive axonemes range between 2 and 4 μm in length, whereas KO axonemes are severely stunted. *C*, quantification of axoneme length. Student’s *t* test analysis of two datasets was performed assuming equal variance. *p* = 3.22946E-10. N = 3. ∗∗∗ *p* ≤ 0.001.

As *Inpp5e*^−/−^ OS development is severely impaired, we next explored how far the axoneme could extend into these altered OS structures. In WT control at P10, the axoneme visualized with an anti-acetylated α-tubulin (Ac-Tub) antibody (red) was extended from the CC (green) for approximately 4 μm (Fig. 12, *A* and *C*). In mutant rods, the axoneme was stunted and never exceeded 1 μm (Fig. 12, *B* and *C*), consistent with transmission electron microscopy results (Fig. 10*D*).

## Discussion

### Photoreceptor cilia are distinct from the primary cilia

In this study, we show that INPP5E localizes to the photoreceptor IS and CC, but not to the OS (Figs. 4 and 5). This is in contrast to the primary cilia of hTert–retinal pigmented epithelium and IMCD3 cell where INPP5E populates the entire primary cilium (5, 6, 16). The major role of INPP5E in the primary cilia is to create a phosphoinositol-4-phosphate (PI4P)-rich ciliary membrane environment by catalyzing the conversion of PIP2 to PI4P, which is essential to regulate the ciliary localization of receptor proteins of the sonic hedgehog signaling pathway. Ciliary targeting of INPP5E in IMCD3 cells was shown to be PDEδ/ARL3 dependent (16). The absence of PDEδ or inhibiting PDEδ activity prevented INPP5E delivery to the cilium of mouse embryonic fibroblasts (17).

Using immunostaining with two different antibodies and serial tangential sectioning with immunoblotting (Fig. 4), we show that INPP5E is a predominantly IS protein. This is consistent with previous reports that INPP5E localizes to the Golgi apparatus in immortalized cell lines (2) and photoreceptors (22). Mouse *Inpp5e* expresses two splice variants, both presumably expressed in photoreceptors, with or without the C-terminal exon containing a CAAX box motif for isoprenylation. The long version of INPP5E is prenylated and membrane-associated, and the short version is predicted to be soluble and cytoplasmic. Both prenylated INPP5E and unprenylated INPP5E (C644A) were shown to localize to the Golgi in Tera-1 testis-derived cell lines, mediated by the N-terminal proline-rich domain (2). In mouse retina, prenylated INPP5E is present at lower levels in the ONL (Fig. 4, *A*, *C*, *E* and *G*), presumably docked to the endoplasmic reticulum for post-translational CAAX box processing (removal of –AAX from the CAAX box and carboxymethylation) (38). The bulk of INPP5E is localized throughout the entire IS. Importantly, INPP5E (most likely the prenylated form) also locates to the CC (Fig. 5, *B*, *D* and *E*). Why and how INPP5E is excluded from the photoreceptor OS is unclear.

### *Inpp5e*^−/−^ phenotype

^ret^*Inpp5e*^−/−^ OSs are stunted beginning at P8 and never reach the normal length. At P10, mutant photoreceptors start to degenerate, rhodopsin mislocalizes in the ONL, and the ONL thickness is reduced (Fig. 3). The mislocalization of rhodopsin at P10 and PDE6 at P12 and later (Fig. 7*B*) is likely a secondary effect of failed OS extension. Cone (Fig. 8*G*) and rod ^ret^*Inpp5e*^−/−^ photoreceptors (Figs. 10 and 11) form an apparently normal length CC but do not extend an axoneme into the OS and do not form discs (Figs. 8*G*, 11 and 12). Presence of the CC and absence of OS axoneme extensions and discs have been observed in a number of animal models lacking OS proteins, including rhodopsin KOs (39, 40), *rd1* mouse (PDE6b null allele) (41), and *rds* mouse (PRPH2 null allele) (42). Presence of CC and absence of OS were also observed in deletions of a number of ciliary proteins, including nephrocystin-5 (NPHP5 or IQCB1) (43), nephrocystin-1 (NPHP1) (44), AHI1 (jouberin) (45), lebercilin (LC5) (46), demonstrating that axoneme extension and disc morphogenesis is a complex process requiring a large number of components. Our analysis of *Inpp5e*^−/−^ localization of several OS-resident proteins did not detect any trafficking abnormalities (Fig. 9), suggesting that INPP5E is dispensable for ciliary targeting of key OS proteins.

### Phosphoinositides and tubby proteins

**Figure 13:**
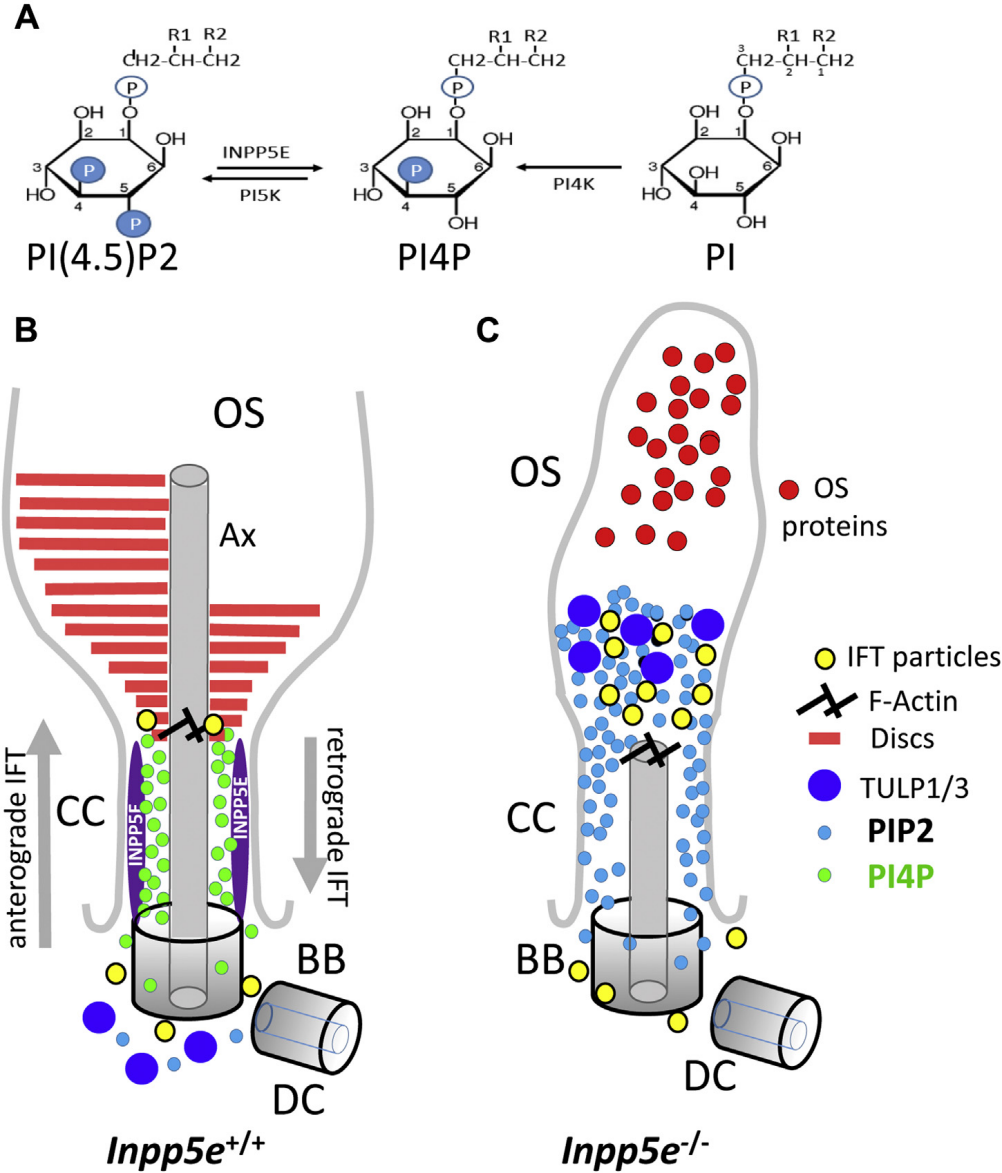
**Model of INPP5E–LCA.***A*, INPP5E removes 5-phosphate at the inositol ring of PIP2 to generate PI4P. R1 and R2 are acyl side chains attached to glycerol. *B*, *cartoon* of a photoreceptor proximal OS with basal body (BB), daughter centriole (DC), and axoneme (Ax). INPP5E is located within the CC and maintains the concentration of local PI4P (*green*). F-actin and actin cytoskeleton components are essential for disc morphogenesis. Anterograde and retrograde IFT (*gray arrows*) are essential for building the axoneme and disc structures (64). *C*, in the absence of INPP5E, PIP2 (*light blue*) accumulates in the CC and spreads into the proximal OS. Excess PIP2 attracts PIP2-interacting proteins TULP1/3 (*deep blue circles*). TULP3 is known to interact with IFT-A particles and may be responsible for accumulation of IFT-A and IFT-B particles atop of the CC, impairing IFT. CC, connecting cilia; IFT, intraflagellar transport; INPP5E, phosphatidylinositol polyphosphate 5-phosphatase; OS, outer segment; PIP2, PI (4,5)P_2_.

The biochemical function of INPP5E is to hydrolyze 5-phosphates at the inositol ring of PIP2 phosphoinositides, generating PI4P (Fig. 13*A*). In photoreceptors, the concentration of phosphatidylinositol polyphosphates in RIS and ROS is very low at roughly 0.04 mol% (47). Recent measurements with fluorescent phosphoinositide sensors revealed that the majority of PI4P and PIP2 in rods is present in the IS and cytoplasm surrounding the nuclei (48); the OS also contains smaller amount of PI4P detectable by MS but no detectable PIP2. The latter is consistent with a direct measurement showing the presence of trace amount of PI4P but not PIP2 in the isolated bovine OS (49). Our results, specifically the localization of INPP5E at the CC (Fig. 4*J*) and misplacement of PIP2-interacing TULP1/3 to the mutant OS (Fig. 9, I and *J*), suggest an involvement of phosphoinositides in axoneme elongation and disc morphogenesis.

Tubby family proteins (Tubby, Tulp1-4) are known to bind PIP2 through their C-terminal “tubby domain” (50). TULP1 was shown to be involved in transport of rhodopsin and is thought to interact with F-actin in photoreceptors (51, 52). TULP3 is required for primary ciliary entry of ARL13b and INPP5E (20) and for ciliary entry of certain GPCRs by bridging the IFT-A complex with PIP2 (53, 54). The increase of TULP3 may explain the accumulation of IFT proteins at the OS base (Fig. 11*D*) as TULP3 also binds to IFT-A (53, 54). Accumulation of PIP2, TULP3, and IFT particles was also observed in the *Inpp5e*^−/−^ primary cilia of neural stem cell cultures with ciliary defects (5, 6).

### Model of INPP5E–Leber congenital amaurosis

We propose a model in which INPP5E is located at the inner leaflet of the CC membrane (Fig. 13*B*) where it controls the composition and levels of PI4P and PIP2 phosphoinositides, as has been observed in primary cilia (5, 6). In WT CC and proximal OS, the concentration of PIP2 is very low due to the activity of INPPP5E. Deletion of INPP5E is expected to increase the concentrations of PIP2 produced by a PI4P-5K kinase. PIP2 infiltrates the CC and accumulates in the proximal OS. Excess PIP2 attracts TULP3 and TULP1 and causes accumulation of retrograde IFT particles at the OS base, thereby causing an IFT impairment. Because anterograde IFT, mediated by kinesin-2 (36), and retrograde IFT, mediated by dynein-2 (55, 56), are essential for OS formation and extension, KO OSs fail to extend their axonemes and form discs. Open questions are whether axoneme extension is necessary for disc formation and whether Tubby protein translocation to the KO OS directly impairs IFT. Future research will explore these questions and determine the levels of PIP2 at the CC and the OS base. It will be essential to further characterize changes in protein composition at this location and, ultimately, determine the exact mechanism preventing disc morphogenesis and axoneme extension.

## Experimental procedures

### Animals

Procedures were approved by the University of Utah Institutional Animal Care and Use Committee and were conducted in compliance with the National Institutes of Health Guide for Care and Use of Laboratory Animals. Floxed *Inpp5e* mice (*Inpp5e*^*F/F*^) were provided mice (Dr Chris Mitchell, Monash University, Australia) and maintained in a 12:12 h dark-light cycle. A transgenic mouse expressing EGFP–CETN2 fusion protein (JAX stock # 008234) was used to identify centrioles/transition zones (57). Three-month-old Long-Evans rats used for tangential retinal sectioning were obtained from Charles River.

### Generation of ^ret^Inpp5^−/−^ conditional KO mice

*Inpp5e*^*F/F*^ mice (12) were mated with Six3-Cre transgenic mice (25) to delete exons 2 to 6, thereby generating retina-specific *Inpp5e* KOs (^ret^*Inpp5e*^−/−^) (Fig. 1). KO mice were viable but had a reduced litter size (4–5 pups). *Inpp5e* floxed allele PCRs were performed on genomic DNA (13) using Inpp5e WT forward primer 5-GAGAAGCTGATAGATGGCTAGG and Inpp5e WT reverse primer 5-AACCAGAAGACCTCATCAAACC and EconoTaq PCR according to manufacturer's specifications (Lucigen corporation) (Fig. 1*D*). Homologous recombination in the retina was verified with INPP5E-KO-F primer 5-CAGAATGCATAGCTCTCTGGGCAAC and INPP5E WT-R primer 5-GTAGTGACATCCCCTGGGCACGTG (amplicon size 450 bp) using retina DNA as a template. The *Inpp5e* floxed allele amplicon is 429 bp, the *Inpp5e* WT allele is 300 bp, and the KO amplicon is 450 bp. Six3-Cre mice were genotyped with Cre-specific primer set Six3Cre159 forward 5-TCGATGCAACGAGTGATGAG and Six3Cre160 reverse primer 5-TTCGGCTATACGTAACAGGG (amplicon size 500 bp). The Egfp-Cetn2+ transgene was identified with the primer set, Egfp-Cetn2+-F 5-TGAACGAAATCTTCCCAGTTTCA and Egfp-Cetn2+-R 5-ACTTCAAGATCCGCCACAACAT (amplicon size 600 bp). PCR amplicons were separated using 1.5% agarose gel electrophoresis in the presence of ethidium bromide and visualized *via* transillumination. The absence of *rd8* and *rd1* mutations was confirmed by PCR (58).

### Immunoblotting

For Western blotting, two retinas from one mouse were homogenized in 100 μl of 50-mM Tris HCl (pH 8), 100-mM NaCl, 10-mM EDTA, and 0.2% Triton X-100, and 2 μl of 100-mM PMSF and 2-μl protease inhibitor. Samples were then sonicated for 2 x 20 pulses at an intensity of 30% and spun at 15,000 rpm for 20 min. The protein concentration was determined by the Bradford assay. Proteins of retina lysates were separated by 10% SDS-PAGE and transferred to a nitrocellulose membrane (59). The resulting membrane was sequentially subjected to blocking for 1 h, primary antibody incubation overnight at 4 °C, and secondary antibody incubation for 1 h. Primary antibodies were diluted 1:500 for anti-INPP5E (PT) and 1:5000 for anti-Rho (4D2). Secondary antibodies were iR680 goat-anti-mouse (1:5000) and iR800 goat-anti-rabbit (1:3000) (Odyssey); images were acquired using an Odyssey scanner. The intensities of protein bands were measured using ImageJ and normalized to control bands.

### Confocal immunohistochemistry

Animals were dark-adapted overnight and sacrificed in ambient light. For conventional fixation (60), P6-P21 eyes from control and mutant mice were immersion-fixed using freshly prepared 4% PFA in 0.1 M phosphate buffer, pH 7.4, for 2 h on ice. Eyecups were then moved to 15% sucrose in phosphate buffer for 1 h and then to 30% sucrose overnight for cryoprotection.

For the low fixation protocol (CO INPP5E antibody and MAK CC marker do not work with standard fixation methods), mouse eyes were rinsed in 1x PBS followed by embedding in optical cutting temperature (OCT). Blocks were immediately frozen at −80 °C and sectioned at 14 um. Slides were removed from freezer and warmed no longer than 5 min. 1% PFA (made by diluting 4% PFA in PBS) was applied to the slide for 2 min. Slides were washed for 5 min in 1x PBS. Sections were blocked using 10% normal goat serum in 0.1 M phosphate buffer–0.1% Triton X-100 for 1 h and incubated with the primary antibody overnight in a rotating humidified chamber at 4 °C.

To show INPP5E localization at the CC *in vivo*, deeply anesthetized P10 mice were transcardially perfused with 7-ml 4% PFA in 0.1 M PBS, pH 7.4, at room temperature (RT) and enucleated eyes were postfixed overnight at 4^o^C. Samples were cryoprotected in 30% sucrose, embedded in optical cutting temperature, and cut into 100-μm sections. Free-floating sections were treated with 1% SDS, 1% β-mercaptoethanol, and 0.1 M PBS (prewarmed to 50 °C) for 10 min at RT. Sections were washed thoroughly with 0.1 M PBS and subject to INPP5E immunostaining (PT antibody, 1:500) with the standard free-floating section method. After overnight incubation, the secondary antibody (Alexa 555-conjugated goat anti-rabbit IgG) was applied at 1 μg/ml and incubated for 1 h at RT. Stained sections were mounted to a slide with a brush, air-dried, and coverslipped with the Fluoromount-G mounting medium (SouthernBiotech).

Cryosections were incubated with the following polyclonal primary antibodies: rod anti-transducin-α (1:500, Santa Cruz); anti-M/L-opsin (1:500, Chemicon); anti-S-opsin (1:500, Chemicon), MOE (anti-rod PDE6, 1:500, Cytosignal); anti-cone PDE6 (1:500, gift of Dr Tiansen Li, NEI); PROM1 (PT) (1: 400); CNGA1/3 (1:1000, NeuroMab, Davis); CDHR1 (1:500, Jun Yang, The University of Utah); SPATA7 (1:100, Dr Rui Chen, Baylor College of Medicine); rat CEP290 (1:300, Dr Anand Swaroop, NEI); IFT88, IFT57, and IFT140 (1:500 Dr Greg Pazour); TULP3 (1:200, PT). mABs included the following: Ac-Tub (1: 1000, Sigma); G8 (1:500, anti-GRK1, Santa Cruz); PRPH2 (2B6) (1:25, Dr Robert Molday, University of British Columbia); GC1 (IS4) (1:1000, Dr Kris Palczewski, UC Irvine); anti-ARL13b (1:200, NeuroMab); anti-cone arrestin (7G6), Dr Peter MacLeish, Morehouse School of Medicine. Cy3- or Alexa488-conjugated goat-anti-rabbit and goat-anti-mouse secondary antibodies were diluted 1:1000 in the blocking solution (2% BSA, 0.1 M phosphate buffer, pH 7.4, containing 0.1% Triton X-100). Images were acquired using a Zeiss LSM800 confocal microscope.

### Retinal serial sectioning with Western blotting

Experiments were performed as described (61, 62). Rats were sacrificed, and eyes were enucleated and dissected in ice-cold Ringer’s solution. A retina punch (3 mm diameter) was cut from the eyecup with a surgical trephine positioned adjacent to the optic disc, transferred onto PVDF membrane with the photoreceptor layer facing up, flat-mounted between two glass slides separated by plastic spacers (ca. 240 μm), and frozen on dry ice. The retina surface was aligned with the cutting plane of a cryostat, and uneven edges were trimmed away. Progressive 5-μm tangential sections were collected, subjected to SDS-PAGE, and probed with antibodies against INPP5E (PT) and rhodopsin (4D2).

### Measurement of the ONL thickness

Mouse eyecups, with the anterior segments and lens removed, were fixed overnight in 4% PFA in PBS overnight. Eyes from both control and mutant mice were then immersed in 15% sucrose in the phosphate buffer for 1 h and then to 30% sucrose overnight for cryoprotection. Twelve-micron transverse sections were stained with 4′,6-diamidino-2-phenylindole, and the thicknesses of the ONL layers were measured along the retinal vertical meridian at approximately 500 μm apart on each side of the optic nerve head.

### Electroretinography

Scotopic and photopic electroretinography (ERG) responses were recorded from P15 WT, ^ret^*Inpp5e*^+/−^ and ^ret^*Inpp5e*^−/−^ mice using a UTAS BigShot Ganzfeld system (LKC Technologies). ERGs were measured as described (22, 63).

After dark adaptation overnight, mice were anesthetized by intraperitoneal injection of ketamine and xylazine (0.1 mg and 0.01 mg/g body weight, respectively), and pupils were dilated with 1% tropicamide. Full-field scotopic retinal electrical responses were recorded in the dark upon a series of white-light stimuli shown below at increasing intensities. Full-field photopic retinal electrical responses were subsequently recorded after light adaptation for 5 min at 33.25 cd s/m^2^. The a-wave amplitude of scotopic ERGs was measured from the baseline to the peak of the cornea-negative wave, and the b-wave amplitude of scotopic and photopic ERGs was measured from the peak of the cornea-negative wave to the peak of the major cornea-positive wave.

### Transmission electron microscopy

Isolated mouse eyecups at ages of P8 and P10 were fixed by immersion in fixative (1.5% glutaraldehyde-1% PFA in 0.1 M cacodylate buffer, pH 7.4) at 4 °C overnight (32, 46). The eyecups were postfixed with 1% osmium tetroxide in 0.1 M cacodylate for 1 h, buffer-rinsed, stained *en bloc* with uranyl acetate, and subsequently dehydrated in an ascending series of methanol solutions. Eyecups were embedded in Epon resin (Ted Pella, Inc) for sectioning. 1 μm plastic sections were cut to face and orient photoreceptors near the optic nerve. Retina ultrathin (60 nm) sections were cut and transferred onto slot grids with carbon-coated Formvar film (Electron Microscopy Sciences) and poststained with uranyl acetate followed by lead citrate. Transmission electron microscopy was performed at 75 kV using a JEOL electron microscope.

### Statistics

SigmaPlot12 was used for statistical analysis using student *t* test and the level of statistical significance was set *p* = 0.05.

## Data availability

All data contained in the manuscript are located within the article.

## Conflict of interest

The authors declare that they have no conflicts of interest with the contents of this article.
